# Evidente—a visual analytics tool for data enrichment in SNP-based phylogenetic trees

**DOI:** 10.1093/bioadv/vbac075

**Published:** 2022-10-12

**Authors:** Mathias Witte Paz, Theresa A Harbig, Kay Nieselt

**Affiliations:** Institute for Bioinformatics and Medical Informatics, University of Tübingen, Tübingen 72076, Germany; Institute for Bioinformatics and Medical Informatics, University of Tübingen, Tübingen 72076, Germany; Institute for Bioinformatics and Medical Informatics, University of Tübingen, Tübingen 72076, Germany

## Abstract

**Motivation:**

A common practice in the analysis of pathogens and their strains is using single-nucleotide polymorphisms (SNPs) to reconstruct their evolutionary history. However, genome-wide SNP-based phylogenetic trees are rarely analyzed without any further information. Including the underlying SNP data together with further metadata on the respective samples in the exploration process can facilitate linking the genomic and phenotypic properties of the samples.

**Results:**

We introduce Efficient VIsual analytics tool for Data ENrichment in phylogenetic TreEs (Evidente), a web-application that provides an interactive visual analysis interface for the simultaneous interrogation of phylogenetic relationships, genome-wide SNP data and metadata for samples of an organism. Besides visualizing the phylogenetic tree, Evidente classifies SNPs as supporting or non-supporting of the tree structures and shows the distribution of both types of SNPs among samples and clades of interest. Furthermore, additional metadata can be included in the visualization. Lastly, Evidente includes an enrichment analysis to identify over-represented genomic features encoded by GO-terms within the clades of the tree. We demonstrate the usability of Evidente with the data of the pathogens *Treponema pallidum* and *Mycobacterium leprae*.

**Availability and implementation:**

Evidente is available at the TueVis visualization web server at https://evidente-tuevis.cs.uni-tuebingen.de/, it can also be run locally.

**Supplementary information:**

Supplementary data are available at *Bioinformatics Advances* online.

## 1 Introduction

**Fig. 1.**
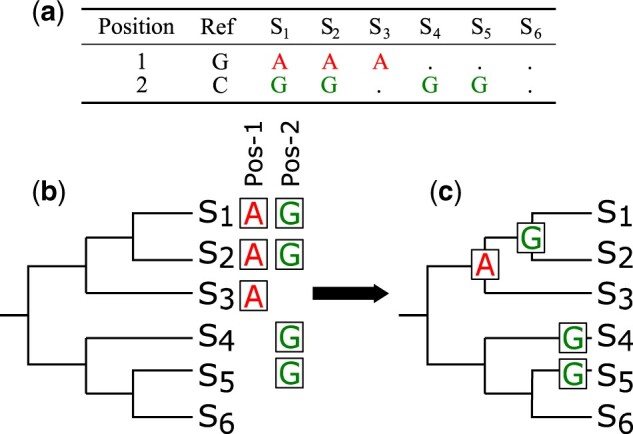
(**a**) Example of a SNP-table with toy data on genomes of six strains. Each row describes a position with SNPs at the strains when compared to the reference allele. A dot character indicates that no mutation appears within the given strain, while any character indicates the presence of a SNP. (**b**) The SNPs at Positions 1 and 2 are aligned to the leaves of the phylogenetic tree. (**c**) When using CLASSICO, the SNP at Position 1 propagates up to the lowest common ancestor (LCA) of *S*_1_, *S*_2_ and *S*_3_. Since the SNP is found only in one clade, it is clade-specific, thus supporting. The SNP found at Position 2 is found after propagation to the LCA of *S*_1_ and *S*_2_, as well as in the clades formed only by *S*_4_ and *S*_5_, respectively. Hence, this SNP is not clade-specific, i.e. non-supporting

The rise of next-generation sequencing (NGS) techniques allows faster and more efficient procedures for the sequencing of genomes (Goodwin *et al.*, 2016). This has not only lead to the sequencing of thousands of different species across all kingdoms but also to the deciphering of the variability of genomes within a species. In particular, the massive sequencing of microbial and viral genomes now offers exciting abilities to query the variability of different strains within a bacterial or viral species. Many fields, in particular that of molecular epidemiology but also the field of ancient pathogenomics [see Spyrou *et al.* (2019) for a review], have made enormous progress and created amazing insight in the evolutionary history of human pathogens. NGS techniques in particular have enabled the study of point mutations called *single-nucleotide polymorphisms* (SNPs) or *variants* depending on the frequencies in the populations (SNPs/SNVs, from this point on SNPs) in strains within a species. SNPs are then often used e.g. to reconstruct the evolutionary relationship of the individuals. For this, a common procedure is to first map the NGS data to a common reference genome, followed by the SNP-calling step using a genotyping method. An alternative approach is to align sample genomes with a reference genome and to identify all positions where a sample differs by a mutation from the reference with respect to the coordinate system of the reference genome. The detected SNPs of all samples can be summarized into one *SNP-table* (see e.g. Fig. 1a). A SNP-table, and hence the underlying identified SNPs, can then be used as a basis for the evolutionary reconstruction to compute a SNP-based phylogenetic tree using, e.g. a maximum parsimony approach.

However, the evolutionary reconstruction does not provide a direct relationship between the reconstructed phylogenetic tree and the underlying SNPs. One way of linking these two datasets and also providing a measurement of accuracy of the tree is to compute the clade-specificity of the SNPs. A clade-specific or clade-unique SNP appears only and in all samples of the clade’s leaf-nodes. Hence, clade-specific SNPs support the choice for the reconstructed phylogeny and therefore can be called supporting SNPs. An example for a clade-specific, i.e. supporting SNP, is the SNP found at Position 1 in Figure 1b. If a SNP appears in many clades, we refer to it as a non-supporting SNP. An example for a non-supporting SNP is the SNP found at Position 2 in Figure 1b.

SNP-based phylogenetic trees are often used for the analysis of pathogens and their strains (Filliol *et al.*, 2006; Foster *et al.*, 2009; Pandya *et al.*, 2009). For example, SNP-based phylogenies were used in the analysis of the spread of the pathogen *Mycobacterium leprae* (Schuenemann *et al.*, 2018) as well as in the analysis of the rise of antibiotic resistance of *Treponema pallidum* (Arora *et al.*, 2016). In these studies, the analysis required the inclusion of the identified SNP data as well as further metadata (e.g. the origin place of the sample or its antibiotic resistance) to identify significant correlations among the datasets. To simultaneously interact with the phylogenetic data, the identified genome-wide SNPs and further metadata within one visualization interface can help in identifying possible correlations. Furthermore, the underlying genomic data are often analyzed from the functional perspective or to classify, e.g. microbial samples after disease outbreaks. A prominent example for this is Nextstrain (Hadfield *et al.*, 2018), a visual analytics platform to track the evolution of viruses. It integrates genome sequence data with phylogenetic reconstruction of the strains and metadata, such as geographic origin or host species. Nextstrain has been instrumental in understanding the evolution and spread of SARS-CoV-2, the virus that has caused the COVID-19 pandemic.

There are many pipelines for the phylogenetic reconstruction from SNP data, such as snpTree (Leekitcharoenphon *et al.*, 2012), PhyloSNP (Faison *et al.*, 2014), SNPhylo (Lee *et al.*, 2014; Yang *et al.*, 2018) or SNVPhyl (Petkau *et al.*, 2017). However, they either do not offer any kind of visualization or they lack essential interactivity for a subsequent exploration process. On the other hand, tools, such as Evolview (Subramanian *et al.*, 2019), iTOL (Letunic and Bork, 2019) or PhyD3 (Kreft *et al.*, 2017), provide the visualization of phylogenetic data with additional metadata for the analyzed samples. Still, these tools do not focus on SNP-based trees and hence they neither provide an intuitive interaction with available SNP data nor allow the identification of enriched molecular features, such as Gene Ontology (GO) terms, in genes with either clade-specific SNPs or non-clade-specific SNPs.

To cover this gap of a visual analytics tool, this article presents Efficient VIsual analytics tool for Data ENrichment in phylogenetic TreEs (Evidente). Evidente enables a simultaneous visualization of genome-wide SNP data and phylogenetic data. Furthermore, Evidente allows to integrate additional metadata on the analyzed samples into the visualization. By providing an interactive visual interface, Evidente facilitates the identification of correlations between the phylogenetic clades, their respective SNPs and metadata. Moreover, Evidente allows the user to run an enrichment analysis on the GO (Ashburner *et al.*, 2000) terms associated with the genes in which SNPs occur.

We demonstrate the effectiveness of our approach through an analysis of two case studies using the pathogens *T.pallidum* and *M.leprae*, and thereby show how Evidente can be used to identify clade-specific SNPs as well as visually identify possible correlations between the genotype and metadata.

## 2 Approach

Before we developed the visual interface of Evidente, we first identified four main tasks for the analysis of SNP data together with metadata in a phylogenetic context in collaboration with two domain experts.

Task *T*_1_**Find clade-specific SNPs**: analyze how well the SNPs support the tree structure since the tree structure itself does not give insights into the underlying SNP distribution. By viewing SNPs specific for a clade users can tell how well it is supported by SNPs.

Task *T*_2_**Analyze the underlying SNP/Metadata distribution of a clade**: it is not always of interest to visualize the SNPs and metadata for all individual strains separately, but summarize the data for a clade of interest to see how often the different alleles appear at a position or how the metadata is distributed.

Task *T*_3_**Link Metadata and non-supporting SNPs**: visually identify correlations that are not supporting the tree. Non-supporting SNPs by definition do not correlate perfectly with the tree structure. However, they might correlate with metadata. For instance, a correlation could hint at a connection between genotype and environmental factors.

Task *T*_4_**Analyze a clade functionally**: find out if any gene functions are over-represented in a clade compared to other clades. For example, one can conduct a GO enrichment analysis to find out if any GO-terms linked to genes with identified SNPs are significantly over-represented in a clade compared to the rest of the tree.

With the design of Evidente, we aim to enable the tasks defined above and allow simultaneous interrogation of phylogenetic and genotype relationships, with additional metadata expressed through colorings. To achieve this, Evidente visualizes a phylogenetic tree that has been computed from a set of samples from a single species together with the underlying SNPs and additional metadata aligned to the leaves of the tree. In the following the design and functionalities offered in Evidente are described in detail.

### 2.1 Data preprocessing and input data

The minimum requirement to run Evidente are two input files, which need to be provided by the user: a SNP-table in tsv-format and a Newick file representing the phylogenetic tree of the same samples as represented in the SNP-table. An example pipeline that computes a SNP-table from NGS data is EAGER (Peltzer *et al.*, 2016) in conjunction with the multi-sample analyzer MUSIAL (https://github.com/Integrative-Transcriptomics/MUSIAL). The columns of the SNP-table (see Fig. 1a) represent each strain or analyzed sample. The rows represent the positions in the genome where a SNP has been found in at least one sample. Each entry containing a nucleotide in IUPAC-code indicates the presence of a SNP in the corresponding position. An *N* character represents an unresolved base. A dot symbol symbolizes that the corresponding sample shares the same base with the reference at the given position.

The phylogenetic tree on the same samples as used for the SNP-table can be computed by any method of choice. For example, we use the SNP data and a maximum parsimony approach for this step. In the two use-cases presented below, this has been done using MEGAX (Kumar *et al.*, 2018).

In addition to the phylogenetic tree and the SNP data, Evidente is able to visualize metadata provided by the user. This information should be encoded in a CSV or a TSV-file, with the columns representing the metadata and the rows containing the name of the analyzed samples. For a correct interpretation and visualization of the metadata features, the second row of the metadata file needs to assign each column to a data type category. For this classification, the data types presented by Munzner (2014) were chosen, and should therefore be labeled as *Categorical*, *Ordinal* or *Numerical*. Evidente orders categorical values by their frequency. Ordinal values are sorted alphabetically by default, though the user can re-sort them in Evidente.

Further optional input files are an annotation file of the reference organism in GFF format and a list of the genes with their corresponding GO-terms. The usage of these files will be explained below in the Section 2.3.3.

### 2.2 Computation of the SNP clade-specificity

The goal of Evidente is to link the phylogenetic data and the genome-wide SNP data by an interactive visualization. Therefore, for each SNP Evidente assesses if it is clade-specific, i.e. the SNP appears only in the respective samples of the clade’s descendant leaf-nodes. For the calculation of the clade-specificity Evidente includes a module called CLASSICO (CLAde-Specific Snp IdentifiCatOr), which computes the distribution of the SNPs from the input files with respect to the lowest common ancestors (LCAs) that define a clade in the phylogenetic tree (Fig. 1c). The algorithm first distributes the SNPs within the reconstructed clades by the following method: the identified SNPs are propagated from the leaves toward the root. An internal node receives a SNP from its children, as long as the SNP is present among all descendants with the same allele. This process is repeated through post-ordering of the nodes up to the root of the tree. It is important to note that some SNPs might not propagate at all and will be allocated only to one of the leaves of the phylogenetic tree. If a SNP is allocated to only one node, meaning it is a clade-specific SNP, then it is labeled as supporting of the tree structure, otherwise as non-supporting. Unresolved bases *N* do not undergo this process but are directly labeled as non-supporting SNPs, since they could be labeled as any base which prevents a clear classification. CLASSICO produces two lists of SNPs, one for the supporting and one for the non-supporting SNPs, which are used for the visualizations in Evidente.

### 2.3 Design and features

In this section, the main visual interface as well as design choices of Evidente is described. To illustrate this, we have chosen an artificial phylogeny and exemplary toy SNP and metadata. The corresponding input data can be found in the Supplementary Material (Supplementary Tables S1 and S2).

#### 2.3.1 Evidente’s graphical user interface

**Fig. 2.**
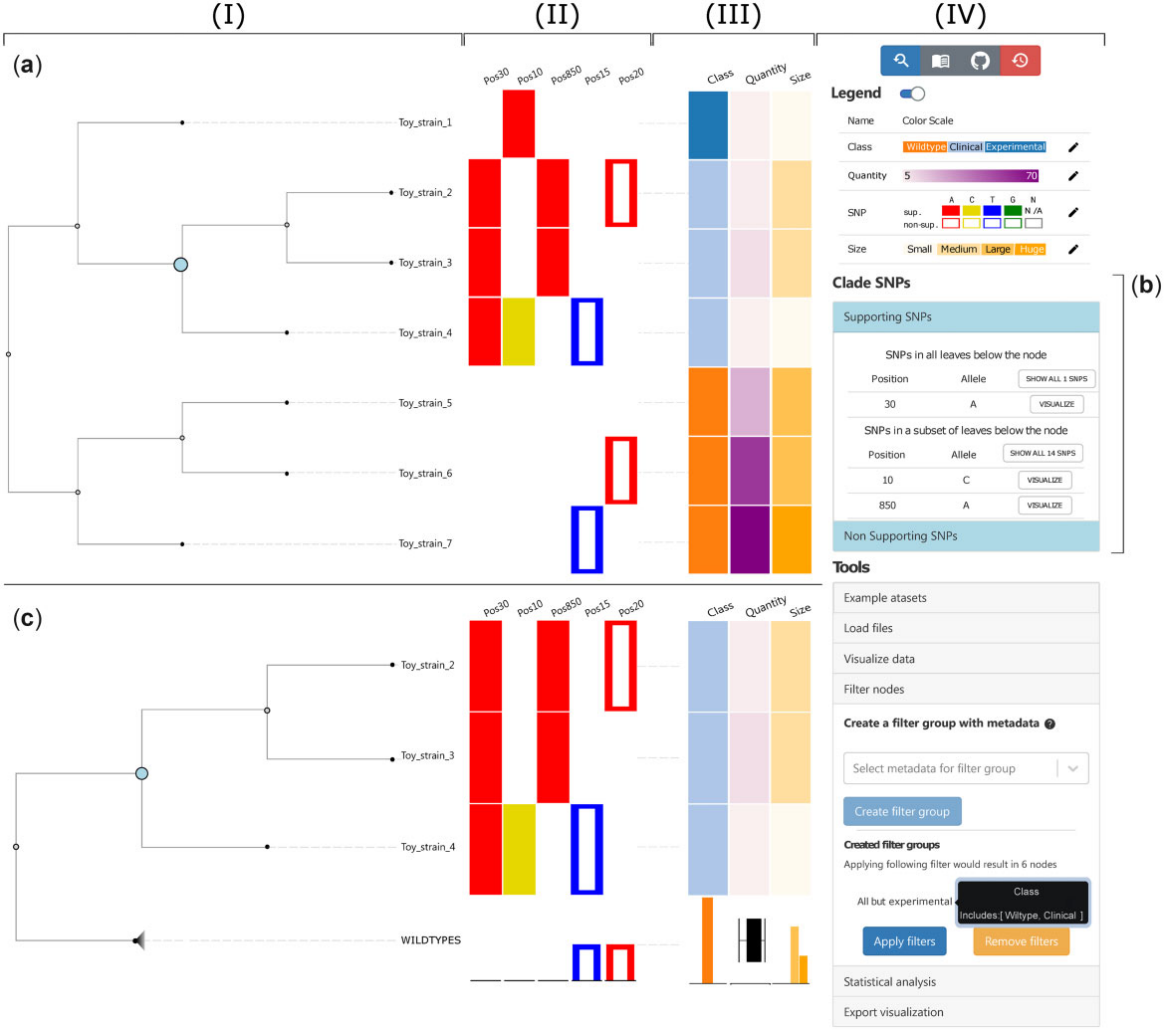
Data visualization using Evidente. The view consists of the main visualization panel (I, II, III) and the sidebar (IV). The main panel visualizes the phylogenetic tree (I), and selected SNPs (II) and metadata (III) as heat maps. The tool’s sidebar (IV) visualizes the legend for the color scales used and further tool menus for the interaction. (**a**) The main visualization shows some SNPs (here: five SNPs from Supplementary Table S1) and metadata (here: three columns from Supplementary Table S2) of the toy dataset. A clade node (emphasized internal node) has been selected. The first SNP (Position 30) corresponds to a supporting SNP that can be found in all leaves of the selected clade, while the supporting SNPs in the next two positions (Positions 10 and 850) appear only in a subset of leaves under the selected clade. The last two visualized SNPs (Positions 15 and 20) are non-supporting SNPs. In a side bar element (**b**) clade SNPs for the currently selected node are summarized and can be added to the visualization. (**c**) Aggregation functionality of Evidente. The subtree containing all samples with the class *Wild-Type* has been aggregated into one node and interactively been renamed as *WILDTYPES*. The end node of a collapsed node is emphasized by a specific glyph (a shaded triangle). The metadata in an aggregated node replaces the heat map cell with box plots (for numerical values) or bar plots for all other data types, including SNP data. Moreover, the filter group ‘All but experimental’ was created as seen in the corresponding menu. It filters out all samples not belonging to the classes *Wild-Type* or *Clinical*. Hence, the strain *Toy_strain_1* and its data have been hidden

The key feature of Evidente is to interactively and simultaneously view and explore a phylogenetic tree and whole-genome SNP data as well as metadata of the samples of one organism. The central part of the graphical user interface of Evidente consists of three visualization components that are horizontally aligned: a horizontal dendrogram and two heat map-like visualizations of SNP and metadata (see Fig. 2I–III). In addition, the user interface includes a sidebar with graphical control elements for access to various functions as well as a settings panel for the color encoding of the SNP data and metadata (see Fig. 2IV).

For the visualization of the phylogenetic tree, we chose a horizontal linear dendrogram. It is created using a modified version of the D3-based library Phylotree (Shank *et al.*, 2018). The resulting interactive dendrogram provides typical interactions for phylogenetic trees, such as selection of branches or nodes, collapsing subtrees on clade-nodes and the hiding of subtrees or leaves. This is described in more detail in Section 2.3.2.

As a horizontal dendrogram was chosen, screen space is used efficiently since heat map-like visualizations of sample-specific data can be aligned horizontally to the leaves (see Fig. 2I). Two separate heat map-like visualizations are aligned to the tree encoding SNPs and metadata using color (see Fig. 2II and III). Separating SNPs and metadata facilitates differentiating the used color scales within both components more easily and helps finding patterns in the SNP data. The heat map visualizing the SNPs shows one position of the genome per column and a rectangle encodes the presence of a SNP in the corresponding sample (see Fig. 2II). The nucleotide of the SNP is visualized using a different color. Cells encoding supporting SNPs receive a full color fill, while non-supporting SNPs are encoded by cells only with a colored frame instead of a fill. Blank spaces encode the absence of a SNP at the given position, i.e. the sample in question showing the reference allele. In the metadata visualization each column of the heat map shows different sample-specific information that can be either of the numerical, ordinal or categorical data type (see Fig. 2III). For numerical data, a continuous sequential color scale was chosen. Similarly, the ordinal data has a sequential color scale as default. Lastly, the categorical values get a default color scale of 20 colors defined by D3 (Bostock *et al.*, 2011). An overview of the color scales of currently displayed data can be found in the legend component (see Fig. 2IV). The color scales can be changed interactively.

Since the distances between leaves, their labels and the heat maps can be too large to easily identify the rows corresponding to the leaves, guiding-lines connect the different visual components. Via hovering interactions of any element, the guiding-lines are highlighted to underline the connection between associated elements. Similarly, vertical guiding-lines are displayed when a user hovers over a cell in one of the heat maps. Furthermore, users can highlight branches in the phylogenetic tree to emphasize their corresponding elements in the heat maps.

#### 2.3.2 Functionalities

Interactive functionalities of Evidente are offered both in the dendrogram itself as well as in the sidebar (see Fig. 2IV). Initially no SNPs or metadata is selected and only the dendrogram is displayed after loading the files. Though the default view uses a dendrogram (i.e. a tree visualization that includes the branch lengths in the visualization), the user can interactively visualize the data as a cladogram, a visualization of the tree that focuses only in the descendent–ancestor relationship without including distances. SNPs belonging to a clade of interest can be explored and added to the view by selecting a node. This interaction is the key for the simultaneous exploration of the SNP and phylogenetic data (*T*_1_). Figure 2a shows such an interaction. After selecting a node, the SNPs corresponding to the clade are listed in a table in the sidebar (Fig. 2b) categorized by their clade-specificity and the presence of the SNP among the leaves of the clade (either present in all leaves or only in a subset of them). The selected node and the respective side bar elements for the clade SNPs are visually linked. Specific SNPs or all SNPs can be selected to be visualized from the clade of interest. Alternatively, using the sidebar, SNPs can be selected by their position and also metadata can be added to the visualization. With the simultaneous visualization of both data sources, it is possible to visually link SNP and metadata patterns (*T*_3_).

Besides the extraction of SNPs for specific clades, further interactions, such as the aggregation and filtering of the data are possible. Any internal node can be collapsed and afterwards labeled as the user desires. This functionality is helpful to get an overview of the characteristics within a summarized clade (*T*_2_) or to simplify the visualization by summarizing clades with similar characteristics into one element depending on their data type. Numerical values are summarized into horizontal box plots, while SNP, categorical and ordinal data are transformed into bar plots. All aggregated plots are scaled to the maximal value within each column to facilitate the comparison of aggregated data. An example for aggregation can be seen in Figure 2c.

On the other hand, the filtering function allows the user to focus the exploration to only a subset of data. For example, when a research question focuses only on a specific strain of the species or that not all available samples have metadata at one’s disposal. There are two alternative filtering strategies for this: either directly from the phylogenetic tree or by using available metadata. For the first option, any node but the root can be selected to be hidden together with all its descendants. The latter option is used from the corresponding section of the side bar by selecting the metadata categories that should be considered for the filtering. For each of the selected categories, the user specifies the desired characteristics of the nodes, i.e. the required range for values within the selected numerical data, or the categories from ordinal or categorical data. With this information the user defines a filter group that can be annotated at will. Every condition within a filter group needs to be fulfilled by a node in order for it to be shown. If many filter groups are created, a node needs to fulfill only one of them in order to be shown. Figure 2c shows an example of the results of a filtering process.

The phylogenetic tree as well as the heat maps allows dragging and zooming both vertically as well as horizontally, which is especially useful for viewing cells of collapsed clades in more detail when a large tree is displayed. The visualizations and legends produced with Evidente can be exported in PNG or PDF format.

#### 2.3.3 GO enrichment analysis

Besides the interactive features mentioned above, Evidente offers an analysis of GO-terms associated with the genes with detected SNPs to identify significantly enriched terms within a clade of the phylogenetic tree (*T*_4_). Before running an enrichment analysis, each SNP that is found within a gene needs to be linked with the GO-terms corresponding to the gene. This is achieved by using the annotation GFF file together with the file containing the GO-terms relationships. Since GO-terms are modeled in a directed acyclic graph (Ashburner *et al.*, 2000), each gene with one or more detected SNPs is not only annotated with the corresponding term but also with all ancestor terms. This propagation is achieved by using the library GOATOOLS (Klopfenstein *et al.*, 2018) and an up-to-date GO hierarchy. The enrichment analysis can be run for a selected clade or for all clades that contain at least one supporting SNP. The enrichment analysis runs independently for each clade as follows: Evidente first extracts all GO-terms that are associated with genes affected by any kind of SNP within the selected clade. For every GO-term a right-sided Fisher’s Exact Test (Fisher, 1922) is conducted. A scheme of the contingency table can be found in Supplementary Table S3. The sum of all cells of the contingency table is equal to the total number of SNPs found within annotated genes. Bonferroni’s method (Holm, 1979) is applied to correct the resulting *P*-values from the statistical tests, using the number of GO-terms identified within the selected clade as the parameter for the correction.

All clades that contain enriched GO-terms after the correction are reported within a *pop-up* modal dialogue with the enriched terms, their description and their respective *P*-value. Using this modal the user is able to highlight the analyzed clade and add SNPs related with each over-represented GO-term to the visualization. Furthermore, the significantly enriched GO-terms can be exported as a CSV file.

#### 2.3.4 Implementation and availability

Evidente is a client–server application that uses established libraries in the backend and the frontend. The frontend of Evidente is written in JavaScript using React (https://reactjs.org/) as a structural framework and the library D3 (Bostock *et al.*, 2011) for the rendering of the visualizations. Evidente uses a python server based on Flask (Grinberg, 2018) for data-parsing and the computation of the enrichment statistics. In order to create the background used for the GO enrichment Evidente makes use of GOATOOLS (Klopfenstein *et al.*, 2018). The module CLASSICO for the calculation of SNP clade-specificity is written in Java and is run as a python subprocess.

Evidente is available on the TueVis server (https://evidente-tuevis.cs.uni-tuebingen.de/), but can also be run locally. The stand-alone version requires NodeJS (Tilkov and Vinoski, 2010) and npm (https://www.npmjs.com/) to be installed.

## 3 Results

The goal of the case studies is to demonstrate how the proposed visual analysis approach enables users to execute the four tasks described in Section 2. For this, Evidente is used to visualize the data of two studies analyzing pathogens: *M. leprae*, which causes Hansen’s disease also known as leprosy (Monot *et al.*, 2009), and *T.pallidum*, which causes the syphilis (subspecies TPA), yaws (subspecies TPE) or bejel (subspecies TEN) disease (Fraser *et al.*, 1998).

**Fig. 3.**
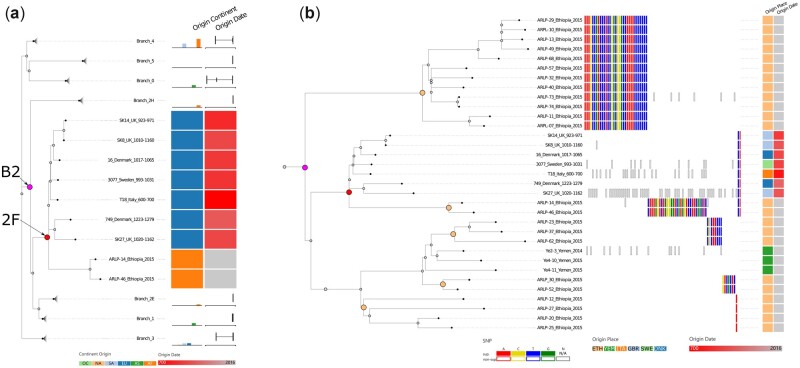
Use-case for Evidente with *M.leprae*’s data presented by Schuenemann *et al.* (2018). (**a**) Visualization of the phylogenetic tree with collapsed branches as presented in the original paper. Only the clade 2*F* (red node) has been left uncollapsed for detailed visualization. (**b**) Detailed analysis of clades found within Branch 2 (B2—purple node). The rest of the clades have been hidden. Using the root of each clade containing only Ethiopian samples (internal nodes emphasized with open circles), the supporting SNPs allocated to them were selected and visualized. The last two SNPs represent those SNPs supporting the clade 2*F* (red node)

The first use-case was performed on the data of the study by Schuenemann *et al.* (2018), which analyzed modern and ancient samples of *M. leprae* extracted from different species around the world. The data consist of a total of 159 samples with 3124 identified SNP positions. The metadata file was manually created with the information provided by the authors in the original publication. To explore the tree with the SNP data and metadata, we used Evidente to first collapse all clades except one clade (labeled 2*F*, see Fig. 3a) according to the branch structures presented in the original paper of Schuenemann *et al.* These branches represent clades of *M.leprae* strains based on different SNP (sub)types. The collapsing allows a detailed inspection of the most ancient clade of *M. leprae*. One of the findings of Schuenemann *et al.* was that the ancient *M.leprae* strains are not present in one common clade, but that they can be found in several branches. Figure 3a shows this distribution by aggregating the metadata. The box plots representing the origin date of the samples indicate that ancient samples also appear in Branches 4, 0 and 3. The bar plots reveal that no clade contains samples from one specific continent of origin. This analysis of the metadata distribution is a typical task in the field, as described in Task *T*_2_.

Next, a specific branch (labeled B2) has been analyzed in detail (see Fig. 3b). The results of the study of Schuenemann *et al.* (2018) suggest that B2 should be split into three different branches (branches 2E/F/H). For a detailed analysis of the branches below B2, all other subtrees were hidden. In order to further analyze the novel branch splits discussed in the paper, the root of clade B2 is selected to identify any SNPs supporting the original split, which is an example for Task *T*_1_ (identifying clade-specific SNPs). For the root of the clade B2, Evidente returns that no SNP is supporting this split. This speaks in favor of the decision of splitting this branch into three different subbranches.

Furthermore, Figure 3b shows that within this clade, Ethiopian samples are not found within a common clade, but are distributed across the subtree. To understand which SNPs support the splits of these clades, we used Evidente to select the root nodes of each clade containing only Ethiopian samples to identify and visualize these SNPs. Figure 3b shows that the first upper two Ethiopian clades have more SNPs supporting this arrangement when compared to the rest of the clades. Finally, we selected the root of the branch 2*F* to identify SNPs supporting this branch that surprisingly consists of two modern Ethiopian samples and many ancient samples. Evidente lists only two supporting SNPs, which underlines the heterogeneity of the strains. These two SNPs could be analyzed in detail to identify their location in the respective genomes and analyze their possible effects.

The data of the second use-case consists of 75 *T. pallidum* samples with a total of 2550 different SNP positions (Pla-Díaz *et al.*, 2022). In addition, we used metadata regarding the antibiotic resistance of some samples against the macrolide azithromycin (Arora *et al.*, 2016) as well as geographical origin, year of isolation and subspecies classification (TPA, TPE or TEN). The original phylogenetic tree contains a long branch that makes the visualization difficult to visualize as a dendrogram. Hence, the data are visualized instead as a cladogram (see Supplementary Fig. S1).

When visualizing the *macrolide resistant* data, Supplementary Figure S1a shows how resistant samples are distributed among the phylogenetic tree. In order to find a possible correlation of this metadata with SNPs, the non-supporting SNPs can be visualized, as stated in Task *T*_3_. By selecting, e.g. the leaf labeled *NE20*, Evidente returns a list of 38 non-supporting SNPs. All SNPs that visually correlate only with the samples with macrolide resistance were kept in the visualization. We tolerated the correlation with unresolved bases (*N*), since they could refer to any allele at the position. Supplementary Figure S1a shows two SNPs having a clear correlation with resistant samples, since all sensitive samples show the reference allele at these positions. These two SNPs are the only ones of the 38 identified SNPs that show a correlation with the resistant samples. These positions (genomic positions 235 204 and 283 649) are found within the 23S rRNA operon, whose modification is associated with the resistance against azithromycin (Stamm, 2010). With this finding, researchers could now test the samples for which the macrolide resistance data are not available but show these SNPs for resistance.

Finally, to demonstrate how Evidente can be used to fulfill task *T*_4_, the clade SS14 of *T.pallidum* was analyzed to identify enriched GO-terms within the clade. The GFF annotation file of the Nichols strain (RefSeq NC_021490) was used, since this reference genome was used to compute the SNP-table (Pla-Díaz *et al.*, 2022). The GO-term IDs related to the coding genes were predicted using FACoP (http://facop.molgenrug.nl/). This analysis returned 61 significantly enriched GO-terms within the SS14 clade. The four most significant results were related to the cell’s membrane (GO: 0061024, GO: 0019867, GO: 0071709, GO: 0009279, *P*-value = 6.29 × 10^−31^). Evidente also allows the user to visualize the distribution of (supporting or non-supporting) SNPs found within genes labeled with the GO-term of interest. Supplementary Figure S1b shows the distribution of all SNPs in those genes that are associated with the GO-term *membrane organization* (GO: 0061024). However, the SNP distribution hints that the over-representation is not unique for clade SS14, since a second clade (with the TPE/TEN samples) also shows SNPs in genes associated with this GO-term.

## 4 Discussion

Evidente is designed to simultaneously visualize the phylogenetic information, genome-wide SNP data and further aspects of an evolutionary reconstruction of different samples or strains from a single species. As a true visual analytics tool, it does not only provide a visual link by aligning the SNPs to the dendrogram, but it also links them computationally with its module CLASSICO, which adds the structural information ‘supporting of the tree structure’ and ‘non-supporting of the structure’ to the SNPs. Moreover, this type of tree visualization is one of the most commonly used in related work and therefore is easy to interpret for users. Another layer of information is added with the GO-term enrichment analysis, which aims to find significantly enriched functions for genes affected by SNPs in a clade of interest. Further features, such as filtering, aggregation and selection, facilitate the interaction with the tool.

With the two case studies, we showed that the simultaneous visualization of SNP data and metadata, and the integration of an enrichment analysis provided in Evidente supports the identification of interesting patterns, within analyses focused on the spread of a pathogen or in antibiotic resistance development. In the first case study, where modern and ancient samples of the *M. leprae* were analyzed, we showed that Evidente can effectively be used to summarize metadata by collapsing clades. Moreover, we analyzed how strongly a certain clade is supported by the SNPs. This distinction helps finding SNPs that are unique to a clade, which might be candidates for a closer inspection by a domain expert.

In the second case study, an exploration process was shown for finding SNPs that are non-supporting for the tree structure but correlate with metadata for samples of *T.pallidum*. Such correlations can, e.g. indicate convergent evolution, where strains in different clades in the tree develop similar mutation due to a shared environmental factor. Furthermore, we were able to show how SNPs in genes associated with a specific GO-term are distributed in the tree to find GO-terms specific for the genes affected by SNPs in two of the three main clades in the phylogenetic tree of *T. pallidum*.

In both case studies, phylogenies of bacterial strains were used. While this data source is suitable since SNP-based phylogenetic trees are often used for the analysis of the evolution of (bacterial but also viral) pathogens and their strains, the interest on eukaryotic pangenomes has started to increase in the last years (Richard, 2020). Due to the simple data formats, Evidente can be applied to any properly formatted phylogenetic tree and SNP data table and thus also deal with eukaryotic pangenomes.

In our use-cases, the phylogenetic tree was based on the SNPs themselves, but it might also be interesting to use the tool for analyzing a tree that is not SNP-based to investigate how well, for instance, a tree reconstructed using the whole genome is supported by SNPs. Currently, the usage of Evidente is restricted to the analysis of samples with a common genomic coordinate system. Hence, insertions and deletions are not considered.

In the current implementation, only SNPs that are found uniquely in clades, i.e. monophyletic groups are considered supporting in order to ensure clade-conformation. However, a further category, which could be considered supporting are SNPs found within a paraphyletic group. Such a third category of SNPs will be a future work for Evidente’s preprocessing module CLASSICO.

Presently, Evidente provides a user-friendly interaction with phylogenetic trees up to a couple of hundred genomes, like the examples shown here on a laptop screen. The largest phylogenetic tree visualized so far in Evidente is the one of *M. leprae*. An overview of the data that consists of 159 samples is provided due to the possibility of collapsing clades. To facilitate the visualization of even larger trees, we plan to work on enhancing the tree scalability. For such trees, one future implementation could provide automatic collapsing of clades to reduce the number of nodes shown. With this, the user is first shown an overview of the data, before having the possibility of decollapsing clades to zoom in and interact with more detailed data, thus implementing the visual information seeking mantra by Shneiderman (1996). With respect to the scalability of the SNPs, as there is a fixed minimum width for the rectangles displaying them, the number of SNPs that can be displayed at the same time depends on the screen width. For the *M. leprae*’s use-case, around 100 SNPs were studied simultaneously. Further SNPs can be explored by horizontally dragging the SNP heat map.

With the inclusion of GO-terms in the analysis, Evidente offers already the exploration of a wide range of functions. Further future work will expand the statistical computations of Evidente, such as e.g. by integrating KEGG pathways as another resource for an enrichment analysis. Moreover, we will provide further methods for statistical correction. In addition, we plan to include the metadata into the computational analysis, by either conducting an enrichment based on the metadata, or by automatically identifying SNP patterns that correlate with metadata.

## 5 Conclusion

We have presented Evidente, a web-application that enables an interactive and simultaneous visualization of phylogenetic, genome-wide SNP data and further metadata. The linked dendrogram of the phylogenetic tree with the two heat map-like table visualizations make the visual assessment of clade-specific or non-clade-specific SNPs and the connection of SNPs with specific metadata as well as enriched GO-terms straight-forward. With these visualization approaches and the functionalities implemented in Evidente, the identified tasks required for the interaction with the underlying data could be addressed successfully as demonstrated in the case studies. Hence, Evidente allows the exploration and identification of possible associations that may not be immediately evident in the data.

## Supplementary Material

vbac075_Supplementary_Data

## Data Availability

The data underlying this article are available in the article and in its online supplementary material.
