# Supplementary material for: Evidente—a visual analytics tool for data enrichment in SNP-based phylogenetic trees
Source: Bioinform Adv. 2022 Oct 12;2(1):vbac075. doi: 10.1093/bioadv/vbac075 (PMC9710622; doi:10.1093/bioadv/vbac075)
Supplement: vbac075_Supplementary_Data [file vbac075_supplementary_data.pdf]

Supplementary material for `Evidente` – A visual analytics tool for data enrichment in SNP-based phylogenetic trees

Mathias Witte Paz, Theresa A. Harbig and Kay Nieselt

September 20, 2022

Table S1: Excerpt of the SNP-table with toy data used for the visualizations of `Evidente`.

**Position**	**Ref**	**Toy_strain_1**	**Toy_strain_2**	**Toy_strain_3**	**Toy_strain_4**	**Toy_strain_5**	**Toy_strain_6**	**Toy_strain_7**
5	A	T	.	.	.	.	T	.
10	G	A	.	.	C	.	.	.
15	A	.	.	.	.	T	.	T
20	T	.	A	.	.	.	A	.
25	C	G	G	G	G	T	T	T
30	C	.	A	A	A	.	.	.
...	...	...	...	...	...	...	...	...

Table S2: Example of the input table for `Evidente` that contains the metadata of the analyzed samples. Each column indicates a metadata feature, while the rows show the strains analyzed. The type of each metadata is specified in the second row named "Type".

**Information**	**Weight**	**Quantity**	**Class**	**Size**
Type	Numerical	Numerical	Categorical	Ordinal
Toy_strain_1	0.17	5	Experimental	Small
Toy_strain_2	0.9	6	Clinical	Medium
Toy_strain_3	0.8	10	Clinical	Medium
Toy_strain_4	0.012	5	Clinical	Small
Toy_strain_5	1100	22	Wild Type	Large
Toy_strain_6	200	55	Wild Type	Large
Toy_strain_7	6000	70	Wild Type	Huge

Table S3: Distributions of all SNPs regardless of their classification that are found within a gene within the contingency table. Each SNP is categorized depending if they are related to the GO-term G and a clade C in question for an over-representation analysis using a right-sided Fisher's Exact Test.

	# SNPs found in C	# SNPs not found in C	
# SNPs related to G	a	b	$a+b$
# SNPs not rel. to G	c	d	$c+d$
	$a+c$	$b+d$	$N = a+b$ $+c+d$

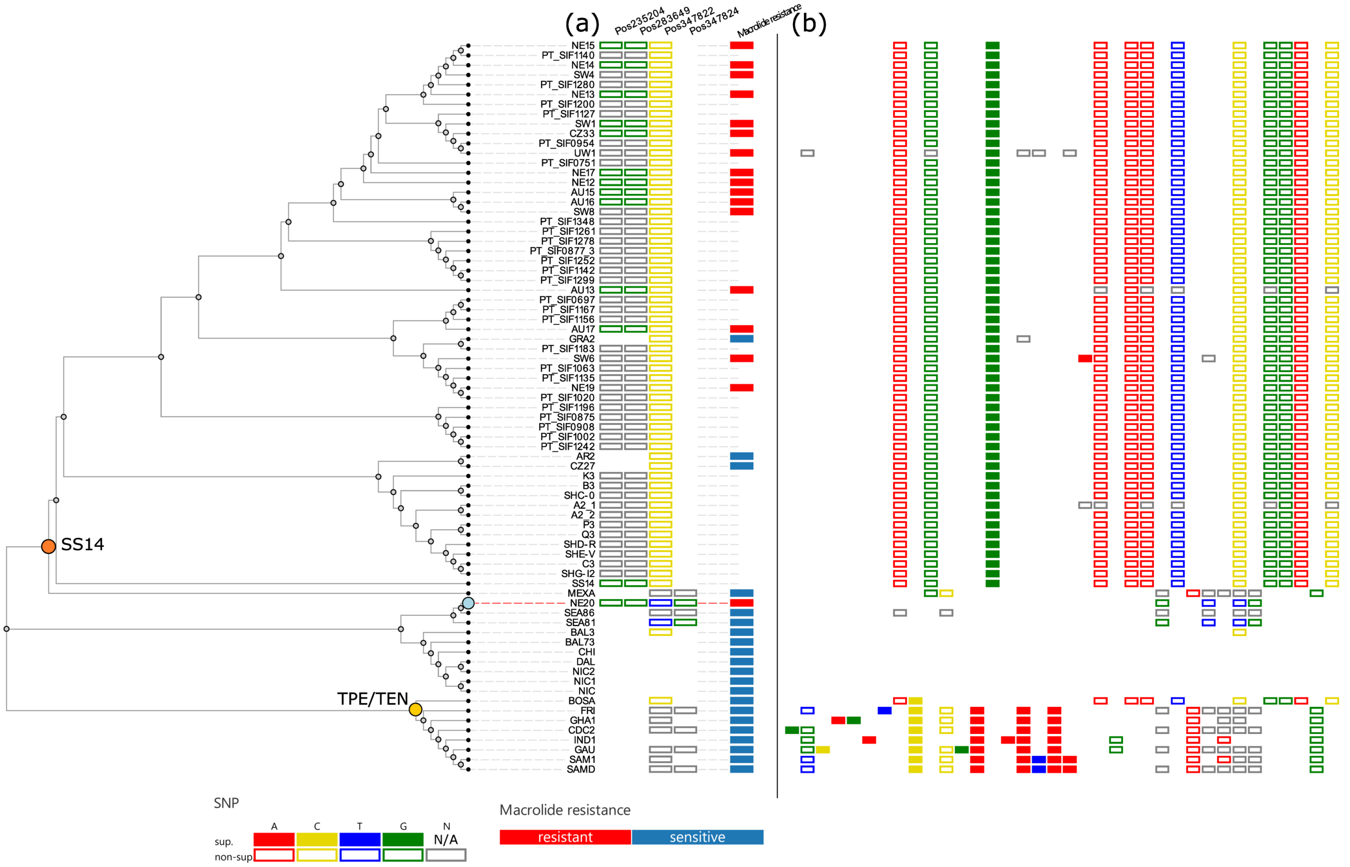

Figure S1: Use case with the *Treponema pallidum* data from a study by Pla-Díaz *et al.* (2022) visualized using a cladogram. (a) Four positions with non-supporting SNPs found in the strain *NE20* are shown together with the visualization on the metadata for *Macrolide resistance*. The first two SNPs show a possible correlation with the samples that are resistant, since every appearance of a SNP occurs only in resistant samples and sensitive samples keep the reference at this position (no SNP visualized). Hence, these SNPs should be considered for a further analysis. For the latter two a correlation can be ruled out, since the SNPs are present in sensitive samples as well. (b) Visualization of all SNPs associated with the GO-term *membrane organization* (GO:0061024). This GO-term was found enriched in the clade SS14 (orange node). However, many SNPs related to this function can also be found within the clade TPE/TEN (yellow node). This suggest that this function could also be enriched in this clade. The positions of the SNPs have been removed for enhanced readability.
